# Cross sectional study on food safety knowledge, attitudes, and practices of food handlers in Lahore district, Pakistan

**DOI:** 10.1016/j.heliyon.2021.e08420

**Published:** 2021-11-17

**Authors:** Muhammad Hashaam Ahmed, Ali Akbar, Muhammad Bilal Sadiq

**Affiliations:** a School of Life Sciences, Forman Christian College (A Chartered University), Lahore, 54600, Pakistan; b Department of Microbiology, Faculty of Life Science, University of Balochistan, Quetta, 87300, Pakistan

**Keywords:** Food safety knowledge, Food safety attitudes, Food safety practices, Food handlers, Food safety legislation

## Abstract

Annually, millions of the people suffer from foodborne diseases which are mainly associated with poor food handling practices. The poor food safety knowledge and practices increase the risk of food contamination and foodborne diseases. The aim of this study was to evaluate the relationship between demographic attributes and food safety knowledge, attitudes, and practices (KAP) of food handlers (chefs and servers) working in small-scale restaurants, hotels and eateries in Lahore, Pakistan. A structured questionnaire including questions related to demographic characteristics and food safety KAP attributes of food handlers (n = 202) was used to collect the responses. The responses of food handlers were statistically analyzed using Spearman Correlation and Chi-Square tests. The results showed that a large proportion of food handlers had good attitudes towards food safety and followed good food safety practices (FSP) but had poor food safety knowledge (FSK). Demographic characteristics of food handlers i.e. level of education, professional category, current job tenure, and total food service industry experience were significantly (p < 0.05) associated with FSK, FSA, and FSP. FSK was found to be moderate to strongly correlated with FSP of food handlers (rs = 0.675), whereas FSA was found to be strongly correlated with FSP (rs = 0.733). The study highlighted the importance and impact of food safety knowledge on food safety practices and overall perspective of food handlers working in restaurants.

## Graphical abstract

**Figure:**
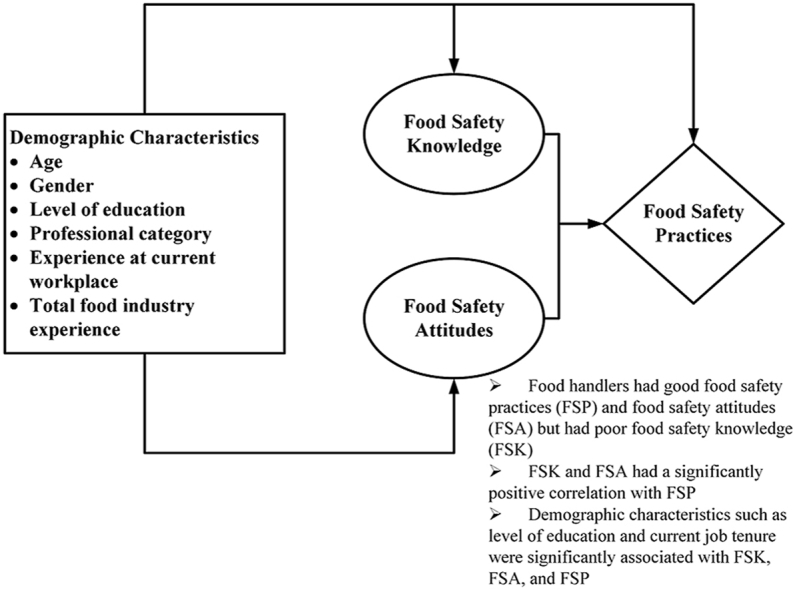

## 1 Introduction

Annually, millions of the people are influenced by foodborne diseases associated with the consumption of contaminated food [1]. Food handlers represent an essential component of commercial food management systems. Food handlers can be responsible for numerous foodborne diseases in case proper food safety practices are not followed [2].

Food safety is directly associated with foodborne diseases and it is essential to improve food safety standards to prevent the spread of foodborne diseases. Foodborne diseases are caused by the consumption of contaminated food items [3]. In developing countries, more than 2 million people die every year from foodborne diseases [4]. This number is expected to increase in the future as food safety has become a worldwide public health issue [5]. The major proportions of the foodborne outbreaks in Europe (61%) and United States (78%) are associated with food consumption from food service establishments [6, 7]. Food safety has become a critical issue worldwide, particularly for developing countries. In order to ensure food safety, food safety knowledge (FSK) is the basic element to start with for enhancing the quality of food safety practices (FSP) being followed. Moreover, food safety knowledge has a significant impact on food safety attitudes (FSA) and practices of food handlers [8]. Improvement in food safety knowledge of food handlers, can ascertain the good food safety practices and measures.

FSK can be defined as the understanding of facts and information related to handling, manufacturing and storing food items, with a primary objective of preventing foodborne diseases and the respective disease outbreaks. It is essential to identify the relationship which exists between FSK and FSP of food handlers as food-related infections are becoming rising concerns globally [9]. FSK is the basic element to start with for enhancing the quality of FSP being followed. Al-Shabib et al. [10], reported that, FSK has a significant impact on food safety attitudes and practices of food handlers.

FSK and FSA of food handlers play an important role in defining their food safety practices. Malpractices during the preparation of food and unhygienic conditions in food preparation areas may cause outbreaks of foodborne diseases [11]. Previously, researchers have analyzed food safety knowledge, attitudes, and practices (KAP) of food handlers to assess the prevailing conditions of food safety in selected regions. Various studies have also associated FSK, FSA, and FSP of food handlers with their demographic characteristics such as age, gender, and level of education, etc [2, 9]. Cempaka et al. [12], concluded in their research study that level of education and food safety practices were significantly correlated.

In developing countries, food safety standards are not strictly followed due to the poor implementation of food safety regulations and poor personal hygiene and practices of food handlers. With an increase in population, the risks associated with foodborne diseases are also increasing. Therefore, it is essential to conduct a food safety survey in developing countries to assess the food safety conditions in restaurants and to propose suitable remedial measures for improvement. Lahore is a metropolitan city of Pakistan and hub of many food industries. Due to recent rapid growth in Lahore, there is an increase in demand of food which resulted in the increase in number food establishments [13]. The aim of this study was to evaluate the food safety knowledge, attitudes and practices of food handlers currently working in different restaurants, hotels, and eateries of two towns within Lahore city of Pakistan. The study also correlated FSK and FSA with FSP of food handlers.

## 2 Materials and methods

### 2.1 Study area

Lahore, the provincial capital of Punjab and the second most populated city of Pakistan, is comprised of 10 administrative divisions (towns). Based on a convenience sampling technique, small-scale restaurants, hotels, and eateries (with 5–10 persons as food handling staff) lying in two towns (Iqbal town and Nishtar town), were selected for the research study. The selected study area can be located on the global map with the coordinates 31.5124° N, 74.2845° E, and 31.4807° N, 74.3505° E, respectively (Supplementary material, Figure S1).

### 2.2 Study design

The research was primarily based on a cross-sectional survey of food handlers (n = 202) by using convenience sampling technique. A total of 300 small-scale restaurants, hotels, and eateries were visited, however only 100 small-scale restaurants, hotels, and eateries agreed to participate in the study. The convenience sampling technique was used due to a very high number of small-scale restaurants, hotels, and eateries at selected study area. The survey was conducted from August 2019 to February 2020 by using a structured questionnaire and informed consent was obtained from all the participants. The questionnaire comprised of 43 questions related to the demographic profile, food safety knowledge, food safety attitudes and food safety practices of food handlers. The data collected in the form of questionnaire responses was analyzed using statistical techniques for the evaluation of food safety KAP scores of food handlers. Food handlers in the research study included chefs and servers at the selected food establishments. The study design was opted from previous research studies conducted on the evaluation of food safety KAP scores of food handlers [10, 14, 15, 16]. The reliability of food safety knowledge questionnaire was evaluated by conducting a pilot scale study on 30 food handlers. Cronbach's alpha for FSK, FSA and FSP questions was 0.850, 0.786 and 0.783, respectively. The questionnaire was modified based on the reliability test results and feedback from two independent food safety experts.

### 2.3 Survey instrument and questionnaire design

The instrument used for the research study was a structured questionnaire. The structure and content of the questionnaire were adapted from past studies [10, 14, 15, 17, 18, 19]. The questionnaire (Supplementary material, questionnaire) comprised of six (06) questions pertaining to the demographic profiles of the respondents, ten (10) questions related to their food safety knowledge, sixteen (16) questions related to their food safety attitudes and eleven (11) questions related to food safety practices. Face to face interviews were conducted for data collection and a methodological framework was developed to study the impact of demographic characteristics, FSK and FSA of food handlers on their FSP. Furthermore, the correlations were also determined, which existed between demographic characteristics of food handlers and their corresponding FSK, FSA, and FSP scores. The data collected through the survey questionnaire was in nominal form. In order to transform the data into a continuous form, all questions (apart from the questions pertaining to demographic characteristics) were assigned scores of either “0” or “1”. The correctness of each answer was determined on the basis of the provincial food authority (Punjab Food Authority, PFA 2017) bylaws and regulations, as mentioned on the regulatory body's official website (http://www.pfa.gop.pk/) and previously published literature [18, 19]. The collected data was encoded in binary format and each correct answer was given a score of 1 whereas each incorrect answer was assigned with a score of 0.

The questionnaire data was encoded (Supplementary material, Figure S2) for food safety knowledge (K), food safety attitudes (A) and food safety practices (P) of food handlers. Eqs. (1), (2), and (3) were used to calculate the total percentage score of a single food handler for food safety knowledge, attitude and practices respectively. The individual food handler scores for FSK, FSA and FSP were in the range of 0–10, 0–16 and 0–11, respectively. The scores were characterized as; below 50% was considered as a poor score, 50–70% as low score with acceptable level and above 70% was considered as good score.

(1) $$\text{Total ​food ​safety ​knowledge ​score}=\frac{K1+K2+\ldots +K10}{10}\;\times 100$$

(2) $$\text{Total ​food ​safety ​attitude ​score}=\frac{A1+A2+\ldots +A16}{16}\;\times 100$$

(3) $$\text{Total ​food ​safety ​practices ​score}=\frac{P1+P2+\ldots +P11}{11}\;\times 100$$

Ethical approval (ERC-78-2019) for this study was obtained from Ethical Review Committee of Forman Christian College (A Chartered University), Lahore, Pakistan.

### 2.4 Data analysis

Statistical analyses were conducted by using SPSS statistical software package (SPSS, version 24.0, USA). Descriptive statistical analyses were performed on the demographic profiles of the respondents, their FSK, FSA, and FSP. The output of descriptive statistics was obtained in the form of frequency and percentage analysis. A p-value less than 0.05 was considered statistically significant.

For the responses related to FSK, FSP and FSA of the food handlers, the mean scores of the participants were calculated. The mean scores of the participants were subjected to Spearman correlation test to determine the strength, nature and significance of the correlations among food safety KAP attributes. Furthermore, the association of demographic characteristics of food handlers with their food safety KAP attributes was studied by Chi-Square test.

## 3 Results and discussion

### 3.1 Demographic characteristics of food handlers

The demographic profiles of the food handlers who participated in the research study are presented in Table 1. The age mix of the food handlers (ranging from under 18 to above 50 years of age) comprised most of the food handlers i.e. 79.2% between the ages of 19–35 years. Old and aged food handlers i.e. 50 years and older, contributed only 2% of the total sample of food handlers whereas, food handlers belonging to the ages of 36–50 years made up to 13.4% of the food handlers' sample. The gender profile of the participants showed 96.5% male and 3.5% of female food handlers’ partaking. Most of the food handlers (32.7%) had undertaken education until high school. The significant proportion (21.3%) of food handlers reported without any formal education. Only 3% of the food handlers obtained technical or vocational education/training and 9.9% of the food handlers reported with university degrees.

**Table 1:** Demographic characteristics of food handlers.

Demographic Characteristic	Variables	Percentage (%)	Frequency (N)
Age	Under 18	5.4	11
	19–35 years	79.2	160
	36–50 years	13.4	27
	50 years and older	2.0	4
Gender	Male	96.5	195
	Female	3.5	7
Level of Education	No formal education	21.3	43
	Primary School	12.4	25
	Middle School	20.8	42
	Technical/Vocational Education	3.0	6
	High School	32.7	66
	University	9.9	20
Professional Category	Cook	25.2	51
	Kitchen Helper	19.8	40
	Server	33.7	68
	Cleaner	8.9	18
	Manager	12.4	25
Current Job Tenure	Less than 1 year	34.2	69
	1–3 years	47.0	95
	3–5 years	10.9	22
	More than 5 years	7.9	16
Total-experience in the foodservice industry	Less than 1 year	10.9	22
	1–3 years	39.6	80
	3–5 years	19.8	40
	More than 5 years	29.7	60

Among categories of respondents, 25.2% of cook, 19.8% of kitchen helpers, 33.7% of food servers, 8.9% of cleaners and 12.4% of managers were reported. Approximately half (47%) of the food handlers who participated in the survey had 1–3 years of professional experience at their current workplaces. The data for “total experience in the food industry” showed that 39.6% of the food handlers had total foodservice industry experience of 1–3 years, 29.7% had professional experience of more than 5 years, 19.8% had an experience of 3–5 years and only 10.9% had an experience of less than 1 year.

### 3.2 Food safety knowledge of food handlers

Food handlers were evaluated for their FSK and it was found that 67.8% of the food handlers never obtained any form of food safety or food handling training, only 5.9% received professional training from a certifying institution and 26.2% of participants received basic informal training (Table 2). Majority of the food handlers (86.8%) accounted improper food handling for foodborne illnesses. The personal experience of 83.2% of participants helped to develop knowledge about foodborne illnesses, 13.4% participants accounted their job trainings whereas, 3% of the participants reported media as a source of information about foodborne illnesses. Among participants, 56.4% of the respondents accounted expired food as a main cause of foodborne illness, whereas, 31.7% of food handlers reported that expired, uncooked and improperly stored foods all together account for foodborne illness. When inquired about the most effective food safety practices in reducing the risk of food contamination, only 33.2% of the food handlers stated that “cleanliness and sanitation in the cooking area, food handlers’ hygiene and using clean water, raw materials and utensils” i.e. all three of the given practices were important. 81.2% of the participants agreed that germs can contaminate food if food safety practices are not exercised. Only 29.7% of the participants reported that germs can contaminate the food through all the factors i.e. poor food handling, unsafe water, uncleaned utensils, lack of cleanliness and sanitation in cooking area. Only 23.3% of the food handlers stated that food wastage, foodborne illness, and damage to food business were all associated with the consumption of unsafe food. Regarding the growth of microbes in food only 43.1% respondents selected correct option, indicating that germs can grow best in warm foods.

**Table 2:** Evaluation of food safety knowledge of food handlers.

Sr #	Question Statement	Variables	Responses (n) %
K1	Have you ever received any training regarding food handling and food safety protocols?	No Training	(137) 67.8 %
		Basic Informal Training	(53) 26.2 %
		Professional Certified Training	**(12) 5.9 %**
K2	Food-borne illnesses can spread through improperly handled, unsafe food.	Yes	**(175) 86.6 %**
		No	(01) 0.5 %
		Not Certain	(26) 12.9 %
K3	What is your source of information about foodborne illnesses?	Personal Experience	(168) 83.2 %
		Job Training	**(27) 13.4 %**
		Media (Print, Electronic, Social)	(06) 3.0 %
		Government Agencies	**(01) 0.5 %**
K4	Which of the following is the most common symptom of foodborne illness?	Diarrhea	**(87) 43.1 %**
		Headache	(05) 2.5 %
		Nausea	(16) 7.9 %
		Vomiting	**(94) 46.5 %**
K5	Which of the following is the most common cause of food-borne illness?	Expired foods	(114) 56.4 %
		Uncooked food	(13) 6.4 %
		Improperly stored food	(11) 5.4 %
		All of the above	**(64) 31.7 %**
K6	Which of the following practice is effective in reducing the risk of food contamination?	Food handlers' hygiene	(15) 7.4 %
		Cleanliness and sanitation in the cooking area	(54) 26.7 %
		Using clean water, raw materials, and utensils	(66) 32.7 %
		All of the above	**(67) 33.2 %**
K7	Germs can contaminate food if food safety practices are not observed.	Yes	**(164) 81.2 %**
		No	(00)) 0.0 %
		Not Certain	(38) 18.8 %
K8	Germs can contaminate food through which of the following ways?	Poor Handling of Food	(40) 19.8 %
		Use of unsafe water and improperly cleaned utensils	(22) 10.9 %
		Lack of cleanliness and sanitation in the cooking area	(80) 39.6 %
		All of the above	**(60) 29.7 %**
K9	The most common effect of consumption of unsafe food is:	Food wastage	(74) 36.6 %
		Foodborne illness	(61) 30.2 %
		Damage to food business	(20) 9.9 %
		All of the above	**(47) 23.3 %**
K10	Germs can grow best in which of the following types of food?	Cold food	(12) 5.9 %
		Hot food	(07) 3.5 %
		Warm food	**(87) 43.1 %**
		Temperature of food has no effect on the growth of germs	(96) 47.5 %

The correct responses were highlighted in bold format. K1-10 indicate food safety knowledge based questions.

Around 86.6% of the participants knew that foodborne illness could spread through improperly handled and unsafe food. The question related to the most common symptom of food-borne illness was answered correctly by 89.6% of the participants. The majority of the food handlers i.e. 68.3% gave incorrect answers when asked about the most common cause of food-borne illness whereas, 66.8% gave incorrect answers related to awareness of food safety practices which must be followed to reduce the risk of food contamination.

### 3.3 Food safety attitudes of food handlers

FSA is an important element of food safety and can effectively control the occurrence of foodborne diseases or hazards [10]. The responses for questions related to FSA of food handlers are summarized in Table 3. Among participants, 76.2% of the participants knew that hot and ready-to-eat food should be kept above 60 °C, 71.3% were aware of the fact that prepared food must be stored below 4 °C in the refrigerator for keeping it safe, 70.3% had a fair idea that preparing food in advance would make it susceptible to the growth of microorganisms. Similar results were reported by Al-Shabib et al. [10], who found that more than 80% of food handlers were aware of the fact that they should not handle the food in case of cuts on fingers and hands and wearing personal staff like jewelry can lead to food contamination. Regarding the safety hazards of smoking in the food preparation area and food handlers with long nails and jewelry, 87.1% of the food handlers were found to be aware of it and 88.6% of food handlers considered handling and learning about food safely as an important part of their job responsibility. Majority of food handlers (93.6%) knew that raw food should be kept separate from cooked food, 98.5% believed that toxic chemicals and cleaning solutions should be placed at a safe distance from the food preparation area and 84.7% participants were of the idea that it was necessary to use antibacterial soap when washing hands. Regarding the defrosting of food items, 48% reported that defrosted foods must not be refrozen.

**Table 3:** Evaluation of food safety attitudes of food handlers.

Sr #	Question Statement	Variables	Responses (n) %
A1	Hot, ready-to-eat food should be kept at a temperature above 60 °C.	Yes	**(154) 76.2 %**
		No	(16) 7.9 %
		Not Certain	(32) 15.8 %
A2	Prepared food should be kept in a refrigerator at 4 °C in order to keep it safe.	Yes	**(144) 71.3 %**
		No	(11) 5.4 %
		Not Certain	(47) 23.3 %
A3	Food is more susceptible to the growth of microorganisms if it is prepared too much in advance.	Yes	**(142) 70.3 %**
		No	(06) 3.0 %
		Not Certain	(54) 26.7 %
A4	It is safe to smoke in an area where food is being prepared.	Yes	(09) 4.5 %
		No	**(176) 87.1 %**
		Not Certain	(17) 8.4 %
A5	Food handlers can have long nails and wear jewelry on their hands. It does not pose any risk to the food being prepared.	Yes	(08) 4.0 %
		No	**(176) 87.1 %**
		Not Certain	(18) 8.9 %
A6	Handling food safely is an important part of my job responsibility.	Yes	**(179) 88.6 %**
		No	(10) 5.0 %
		Not Certain	(13) 6.4 %
A7	Learning more about food safety is important to me and it can help me do my job better.	Yes	**(179) 88.6 %**
		No	(08) 4.0 %
		Not Certain	(15) 7.4 %
A8	The health status of workers should be evaluated before employment.	Yes	**(129) 63.9 %**
		No	(02) 1.0 %
		Not Certain	(71) 35.1 %
A9	Raw food should be kept separate from cooked food.	Yes	**(189) 93.6 %**
		No	(04) 2.0 %
		Not Certain	(09) 4.5 %
A10	Toxic chemicals and cleaning solutions should be stored at a safe distance from the food preparation area.	Yes	**(199) 98.5 %**
		No	(03) 1.5 %
		Not Certain	(00) 0.0 %
A11	Defrosted food should not be refrozen.	Yes	**(97) 48.0 %**
		No	(18) 8.9 %
		Not Certain	(87) 43.1 %
A12	Temperatures of refrigerators and freezers should be checked at regular intervals.	Yes	**(107) 53.0 %**
		No	(03) 1.5 %
		Not Certain	(92) 45.5 %
A13	Food handlers with abrasion or cuts on hands should not touch unwrapped food.	Yes	**(172) 85.1 %**
		No	(05) 2.5 %
		Not Certain	(24) 11.9 %
A14	Employees suffering from an illness should not be permitted to work in the food preparation area.	Yes	**(154) 76.2 %**
		No	(10) 5.0 %
		Not Certain	(38) 18.8 %
A15	It is necessary to use antibacterial soap when washing hands.	Yes	**(171) 84.7 %**
		No	(15) 7.4 %
		Not Certain	(16) 7.9 %
A16	Storage of food in refrigerators kills harmful microbes or germs.	Yes	(57) 29.2 %
		No	**(37) 18.3 %**
		Not Certain	(108) 53.5 %

The correct responses were highlighted in bold format. A1-16 indicate food safety attitude-based questions.

Most of (71.3%) the food handlers had accurate knowledge regarding the storage of prepared foods and 53% were correct in the opinion that temperatures of refrigerators and freezers be checked at regular intervals. About 63.9% of food handlers gave positive and correct replies when asked regarding checking the health status of food handlers whereas, 85.1% of food handlers scored correctly when asked questions related to the working of food handlers with abrasion and cuts. Furthermore, 76.2% of food handlers answered correctly regarding a restriction on the entrance of employees suffering from illness in food preparation areas. Codex Alimentarius Commission [20] described that food handles who are sick or suspected of having a disease which can be transmitted by food should not be permitted to work in food premises. The majority of the food handlers were aware of the fact that during illness they should not be handling the food.

One anomaly within this section was the part where around 71.8% of the participants provided the wrong answer when asked about the storage of foods in refrigerators for controlling the growth of microbes.

### 3.4 Food safety practices of food handlers

The responses of food handlers regarding FSP are summarized in Table 4. More than half of the respondents (57.9%) reported that they washed their hands before cooking or serving food, 54% of the food handlers said that they washed their hands with antibacterial soap every time, 49.5% of the food handlers responded that it is necessary to wash utensils and equipment before food preparation and 91.1% of the food handlers used separate utensils for raw and cooked foods. Around 80.7% of food handlers stated that they check expiry dates on food products before using them, however, 72.8% of food handlers were unaware of standard food thaw practices and reported to thaw frozen food at room temperature.

**Table 4:** Evaluation of food safety practices of food handlers.

Sr #	Question Statement	Variables	Responses (N) %
P1	I always wash my hands before cooking or serving food.	Yes	**(117) 57.9 %**
		No	(66) 32.7 %
		Not Certain	(19) 9.4 %
P2	Do you wash your hands with antibacterial soap?	Yes	**(109) 54.0 %**
		No	(27) 13.4 %
		Occasionally	(66) 32.7 %
P3	Do you wash food contact surfaces such as chopping boards, tables, and knives with antibacterial soap before food preparation?	Yes	**(100) 49.5 %**
		No	(23) 11.4 %
		Occasionally	(79) 39.1 %
P4	Do you use separate kitchen utensils for raw and cooked food?	Yes	**(184) 91.1 %**
		No	(07) 3.5 %
		Occasionally	(11) 5.4 %
P5	Do you continue working when you are sick?	Yes	(18) 8.9 %
		No	**(59) 29.2 %**
		Occasionally	(125) 61.9 %
P6	Do you thaw food at room temperature?	Yes	(147) 72.8 %
		No	**(11) 5.4 %**
		Occasionally	(44) 21.8 %
P7	Do you check the expiry dates of food products before using them?	Yes	**(163) 80.7 %**
		No	(09) 4.5 %
		Occasionally	(30) 14.9 %
P8	Do you check the integrity of food packages before using food products?	Yes	**(126) 62.4 %**
		No	(13) 6.4 %
		Occasionally	(63) 31.2 %
P9	Do you wear a uniform while handling food?	Yes	**(154) 76.2 %**
		No	(43) 21.3 %
		Occasionally	(05) 2.5 %
P10	How often do you change and wash the uniform you use while working?	Daily	**(96) 47.5 %**
		Twice a week	(69) 34.2 %
		Once a week	(06) 3.0 %
		Uncertain	(31) 15.3 %
P11	Do you use disposable tissues when coughing or sneezing and then immediately wash hands?	Yes	**(69) 34.2 %**
		No	(67) 33.2 %
		Occasionally	(66) 32.7 %

The correct responses were highlighted in bold format. P1-11 indicate food safety practices-based questions.

Majority of food handlers (70.8%) kept on coming to their workplaces even when sick thereby yielding negative scores in this respect. With regards to the thawing of frozen food items, only 5.4% of food handlers followed the correct food safety practice of not thawing foods at room temperature. Around 76.2% of workers followed the right practice of wearing uniforms at food preparation areas; however, only 47.5% of the food handlers washed their uniforms regularly. Lastly, only 34.2% of food handlers followed the correct food safety practice of using tissues when coughing or sneezing and then immediately washing hands. Similar results were reported by Kunadu *et al.* [21] and Sneed et al. [22], who found that FSP of food handlers were poor.

The scores for personal hygiene such as washing hands and utensils before food handling were lower than the previous reports [10, 14, 15]. Poor hygiene practices and improper handling of food are the major cause of foodborne illnesses. The washing of hands at every food production step, particularly before handling the food should be exercised by all food handlers [23].

### 3.5 Descriptive statistics for KAP score of food handlers

Food handlers scored a minimum score of “1” and a maximum score of “10” out of 10 in the questionnaire section related to food safety knowledge. The mean score for food safety knowledge was 4.38 ± 2.25 (43.8%) which was considered as poor score (below 50%). The food safety attitudes scores of the food handlers varied between “1” to “16” out of the total score of 16. The mean score for food safety attitudes was found to be 12.00 ± 4.00 (75%) which as considered as a good score. The food handlers attained a minimum food safety practices’ score of “0” and the maximum score of “11” out of 11. The mean score of food safety practices was 5.88 ± 3.02, which was characterized as low score (50–70%).

Dudeja *et al.* [23], showed in their study that participating food handlers were having good FSK, FSA, and FSP scores. During a study based on evaluation of KAP attributes of food handlers in Saudi Arabia, satisfactory FSK, FSA, and FSP scores were observed by Al-Shabib *et al.* [10]. Sani & Siow [24], conducted food safety KAP based survey in Malaysia and found food handlers with good FSK, FSA and FSP scores where the most contributing healthy food safety practices were washing of hands with antibacterial soaps and wearing of gloves before contact with food items. Abdul-Mutalib *et al.* [14] also reported that the food handlers in Malaysia had satisfactory FSK, FSA, and FSP scores whereas, Nee & Sani [25] reported that food handlers in Malaysia had good FSK and understating regarding the importance of hygiene; however, they had poor knowledge scores on food storage practices.

### 3.6 Correlation among food safety knowledge, attitudes and practices of food handlers

Spearman correlation analysis was carried out between FSK and FSP (Supplementary material, Table S1). According to Akoglu [26], a correlation is moderate to strong if Spearman's rho is greater than 0.600 i.e. r_s_ > 0.600. FSK was found to be moderate to strongly correlated with food safety practices of food handlers (r_s_ = 0.675). Moreover, the correlation was found to be significant (p < .05). Spearman correlation analysis was also carried out between FSA and FSP scores of the food handlers. The results demonstrated that the FSA was found to be strongly correlated with FSP (r_s_ = 0.733) and the correlation was found to be significant (p < .05). The significant positive correlation indicates that FSK and FSA of food handlers will influence their FSP. Ansari-Lari et al. [15], reported a significant positive correlation between FSK and FSA of food handlers. The education of personal hygiene alone is not enough to ensure the accurate applications of FSK, FSA and FSP. A hands-on trainings and frequent training programs are essential to improve the overall status of food safety [13, 14].

### 3.7 Association between demographic characteristics and food safety KAP attributes

For assessing the association (p value) of demographic characteristics of food handlers with their food safety KAP attributes, a chi-square test was conducted. There was no significant association between “age” and “food safety knowledge” of food handlers (p = 0.058). However, all other demographic characteristics presented a significant association (p < 0.05) with FSK of food handlers (Table 5). Chi-square test was also carried out between the demographics of participants and their FSA; the results showed that “age” and “gender” were found to be insignificantly correlated with “FSA” with p value of, p = 0.052 and p = 0.161 respectively. Whereas, all other demographic characteristics (level of education, professional category, current job tenure and job experience) were significantly (p < 0.05) associated with FSA. Chi-square test further revealed that except “gender” of food handlers, all the other demographic attributes were found to be significantly associated with “FSP” of food handlers.

**Table 5:** Chi-square test for association between demographic characteristics and food safety KAP attributes.

Demographic Variables	Food Safety Knowledge *p* value	Food Safety Attitude *p* value	Food Safety Practices *p* value
Age	0.058	0.052	0.027
Gender	0.006	0.161	0.717
Level of Education	0.000	0.000	0.000
Professional Category	0.000	0.002	0.001
Current Job Tenure	0.000	0.015	0.004
Total Food Service Industry Experience	0.001	0.000	0.003

The results of the research study showed that age had no significant association with the FSK of food handlers. FSA also had no significant correlation with the age of the food handlers. Among KAP food safety attributes, only FSP had a significant association with the age of participants. Majority of the participants (79.2%) were in the age range of 19–35 years and age of food handlers showed significant association with FSP. Young food handlers (26–35 years) in Ireland were found more knowledgeable about standard food safety practices, which might be due to more exposure of young food handlers and their commitment towards learning food practices [27].

Sanlier & Konaklioglu [28], evaluated the KAP attributes of food handlers in Turkey and reported a significant difference among the FSK, FSA and FSP of male and female participants. However, Patil et al. [29], reported that men were found with poor FSP. McIntyre *et al.* [30], conducted a KAP attribute study in Canada and revealed that there was no association between FSK of food handlers and their gender which corroborated the findings of current research. Since this study involved an overwhelming majority of male food handlers, it could not be concluded how gender affects food safety KAP attributes among food handlers in Pakistan.

FSK was most strongly associated with the level of education of food handlers followed by FSA and FSP. A research study carried out in Vietnam by Vo *et al.* [31], concluded that the level of education had a positive impact on FSK and FSP of food handlers. Soares et al. [16] reported that the level of education was significantly associated with the FSK of food handlers.

In a food safety survey conducted in Brazil by Soares *et al.* [18], education was found to be highly correlated with the FSK of participants. Similar results were reported by McIntyre *et al.* [30] in KAP model-based research survey conducted on food handlers in Canada. Regarding the KAP scores of food handlers, Yarrow et al. [32] ascertained that an increased level of education resulted in better KAP scores. Hence, this research study concludes that the level of education of food handlers have a significant association with KAP based food safety attributes.

## 4 Conclusion

This study concludes that the food safety attitudes and practices of food handlers were satisfactory, however, food safety knowledge was poor. All demographic attributes except age were significantly associated with food safety knowledge. Except age and gender all the demographic characteristics (level of education, professional category, current job tenure and job experience) were significantly (p < 0.05) associated with FSA. All the demographic attributes (age, level of education, professional category, current job tenure and job experience) except gender were found to be significantly associated with FSP of food handlers. A significant (p < .05) positive correlation was observed between FSK and FSP of food handlers. Similarly, the correlation between FSA and FSP was significant (p < .05). The findings of this study affirm that food safety knowledge of the food handlers should be improved. The overall, KAP attitudes of food handlers can be improved by indulging the food establishments into various food safety training and encouragements which in turn minimize the foodborne outbreaks and food safety threats. Food safety knowledge and attitudes are closely interconnected therefore, future research on the nature of correlation between these food safety attributes with large sample size will help to improve the status of food safety in developing countries. Moreover, future research should be carried out to evaluate the effect of coronavirus disease 2019 (COVID-19) on the food safety knowledge, practices and attitudes of food handlers.

## Declarations

### Author contribution statement

Muhammad Hashaam Ahmed: Conceived and designed the experiments; Performed the experiments; Analyzed and interpreted the data; Contributed reagents, materials, analysis tools or data; Wrote the paper.

Ali Akbar: Analyzed and interpreted the data; Contributed reagents, materials, analysis tools or data; Wrote the paper.

Muhammad Bilal Sadiq: Conceived and designed the experiments; Analyzed and interpreted the data; Contributed reagents, materials, analysis tools or data; Wrote the paper.

### Funding statement

This research did not receive any specific grant from funding agencies in the public, commercial, or not-for-profit sectors.

### Data availability statement

Data included in article/supplementary material/referenced in article.

### Declaration of interests statement

The authors declare no conflict of interest.

### Additional information

No additional information is available for this paper.
