# Supplementary material for: Cross sectional study on food safety knowledge, attitudes, and practices of food handlers in Lahore district, Pakistan
Source: Heliyon. 2021 Nov 17;7(11):e08420. doi: 10.1016/j.heliyon.2021.e08420 (PMC8606342; doi:10.1016/j.heliyon.2021.e08420)
Supplement: Supplement_Material [file mmc1.docx]

**Supplementary material**

**Evaluation of food safety knowledge, attitudes, and practices of food handlers in Lahore district, Pakistan**

**Muhammad Hashaam Ahmed^a^, Ali Akbar^b^, Muhammad Bilal Sadiq^a,*^**

^a^School of Life Sciences, Forman Christian College (A Chartered University), Lahore, 54600, Pakistan.

^b^Department of Microbiology, Faculty of Life Science University of Balochistan Quetta, 87300, Pakistan

*Authors for correspondence:

*Muhammad Bilal Sadiq; School of Life Sciences, Forman Christian College (A Chartered University), Lahore, 54600, Pakistan. Tel: +92 (0) 336-0416900

Email: [m.bilalsadiq@hotmail.com](mailto:m.bilalsadiq@hotmail.com)

<https://orcid.org/0000-0003-2487-0468>


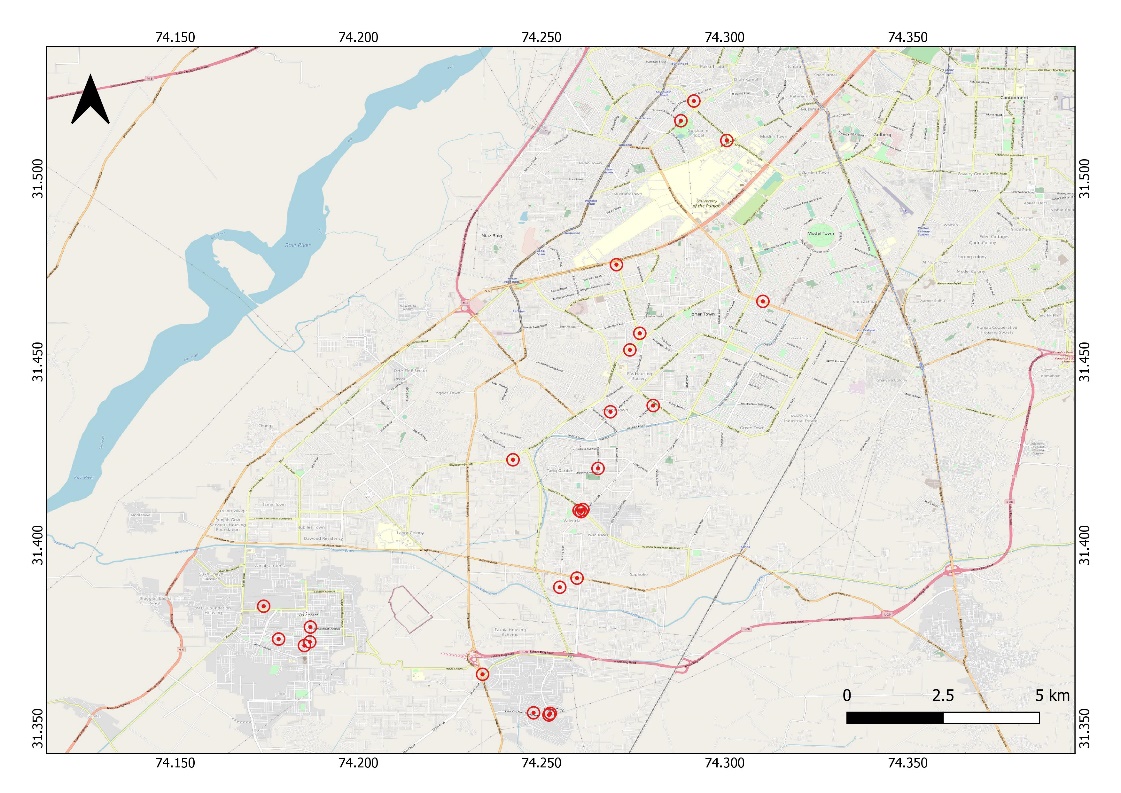


**Figure S1** Research study map (red dots indicate the regions from where the food handlers were sampled for the research study)


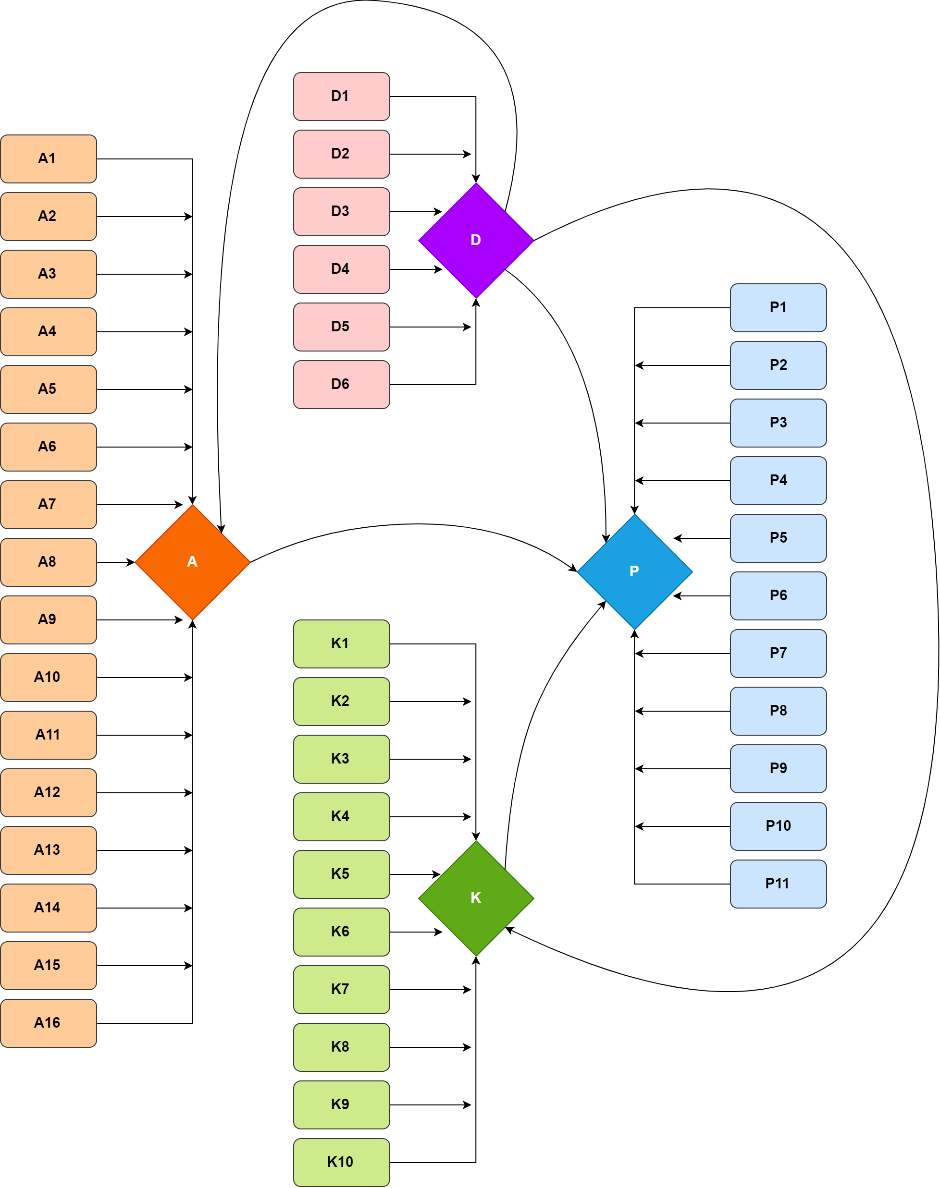


**Figure S2** Encoded questionnaire, where K, A and P indicate knowledge, attitude and practices respectively.

**Table S1** Correlation among food safety KAP attributes

| Level | Spearman's rho | p |
| --- | --- | --- |
| Food Safety Knowledge-Food Safety Practices | 0.675 | 0.000* |
| Food Safety Attitudes-Food Safety Practices | 0.733 | 0.000* |

**Detailed survey questionnaire (Correct responses were highlighted in green color)**

D1) What is your Age?

- Under 18
- 19 - 35 years
- 36 - 50 years
- 50 years and older

D2) What is your Gender?

- Male
- Female

D3) What is your Level of Education?

- No formal education
- Primary School
- Middle School
- Technical/Vocational Education
- High School
- University

D4) Which Professional Category are you in?

- Cook
- Kitchen Helper
- Server
- Cleaner
- Manager

D5) How much experience do you have at your current workplace?

- Less than 1 year
- 1 to 3 years
- 3 to 5 years
- More than 5 years

D6) How much total experience do you have in the food service industry?

- Less than 1 year
- 1 to 3 years
- 3 to 5 years
- More than 5 years

K1) Have you ever received any training regarding food handling and food safety protocols?

- No Training
- Basic Informal Training
- Professional Certified Training

K2 Food-borne illnesses can spread through improperly handled, unsafe food.

- Yes
- No
- Not Certain

K3) What is your source of information about foodborne illnesses?

- Personal Experience
- Job Training
- Media (Print, Electronic, Social)
- Government Agencies

K4) Which of the following is the most common symptom of foodborne illness?

- Diarrhea
- Headache
- Nausea
- Vomiting

^K5) Which of the following is the most common cause of foodborne illness?^

- Expired foods
- Uncooked food
- Improperly stored food
- All of the above

K6) Which of the following practices is most effective in reducing the risk of food contamination?

- Food handlers' hygiene
- Cleanliness and sanitation in the cooking area
- Using clean water, raw materials, and utensils
- All of the above

^K7) Germs can contaminate food if food safety practices are not observed.^

- Yes
- No
- Not Certain

K8) Germs can contaminate food through which of the following ways?

- Poor Handling of Food
- Use of unsafe water and improperly cleaned utensils
- Lack of cleanliness and sanitation in the cooking area
- All of the above

K9) The most common effect of consumption of unsafe food is:

- Food wastage
- Foodborne illness
- Damage to food business
- All of the above

K10) Germs can grow best in which of the following types of food?

- Cold food
- Hot food
- Warm food
- The temperature of food has no effect on the growth of germs

A1) Hot, ready-to-eat food should be kept at a temperature above 60°C.

- Yes
- No
- Not Certain

A2) Prepared food should be kept in a refrigerator at 4°C in order to keep it safe.

- Yes
- No
- Not Certain

A3) Food is more susceptible to the growth of microorganisms if it is prepared too much in advance.

- Yes
- No
- Not Certain

A4) It is safe to smoke in an area where food is being prepared.

- Yes
- No
- Not Certain

A5) Food workers can have long nails and wear jewelry on their hands. It does not pose any risk to the food being prepared.

- Yes
- No
- Not Certain

A6) Handling food safely is an important part of my job responsibility.

- Yes
- No
- Not Certain

A7) Learning more about food safety is important to me and it can help me do my job better.

- Yes
- No
- Not Certain

A8) The health status of workers should be evaluated before employment.

- Yes
- No
- Not Certain

A9) Raw food should be kept separate from cooked food.

- Yes
- No
- Not Certain

A10) Toxic chemicals and cleaning solutions should be stored at a safe distance from the food preparation area.

- Yes
- No
- Not Certain

A11) Defrosted food should not be refrozen.

- Yes
- No
- Not Certain

A12) Temperatures of refrigerators and freezers should be checked at regular intervals.

- Yes
- No
- Not Certain

A13) Food workers with abrasion or cuts on hands should not touch unwrapped food.

- Yes
- No
- Not Certain

A14) Employees suffering from an illness should not be permitted to work in the food preparation area.

- Yes
- No
- Not Certain

A15) It is necessary to use antibacterial soap when washing hands.

- Yes
- No
- Not Certain

A16) Storage of food in refrigerators kills harmful microbes or germs.

- Yes
- No
- Not Certain

P1) I always wash hands before cooking or serving food.

- Yes
- No
- Not Certain

P2) Do you wash your hands with antibacterial soap?

- Yes
- No
- Occasionally

P3) Do you wash food contact surfaces such as chopping boards, tables, and knives with antibacterial soap before food preparation?

- Yes
- No
- Occasionally

P4) Do you use separate kitchen utensils for raw and cooked food?

- Yes
- No
- Occasionally

P5) Do you continue working when you are sick?

- Yes
- No
- Occasionally

P6) Do you thaw food at room temperature?

- Yes
- No
- Occasionally

P7) Do you check the expiry dates of food products before using them?

- Yes
- No
- Occasionally

P8) Do you check the integrity of food packages before using food products?

- Yes
- No
- Occasionally

P9) Do you wear a uniform while handling food?

- Yes
- No
- Occasionally

P10) How often do you change and wash the uniform you use while working?

- Daily
- Twice a week
- Once a week
- Uncertain

P11) Do you use disposable tissues when coughing or sneezing and then immediately wash hands?

- Yes
- No
- Occasionally
